# A risk score for prediction of poor treatment outcomes among tuberculosis patients with diagnosed diabetes mellitus from eastern China

**DOI:** 10.1038/s41598-021-90664-y

**Published:** 2021-05-27

**Authors:** Nannan You, Hongqiu Pan, Yi Zeng, Peng Lu, Limei Zhu, Wei Lu, Qiao Liu, Leonardo Martinez

**Affiliations:** 1 Department of Chronic Communicable Disease, Center for Disease Control and Prevention of Jiangsu Province, Nanjing, Jiangsu Province China; 2 The Second Affiliated Hospital of Zhejiang, Chinese Medical University, Hangzhou, Zhejiang Province China; 3 Department of Tuberculosis, The Third People’s Hospital of Zhenjiang Affiliated To Jiangsu University, Zhenjiang, Jiangsu Province China; 4 Department of Tuberculosis of Three, Nanjing Public Health Medical Center, Nanjing Second Hospital, Nanjing Hospital Affiliated to Nanjing University of Traditional Chinese Medicine, Nanjing, Jiangsu Province China; 5 Department of Epidemiology, School of Public Health, Boston University, Boston, MA USA

**Keywords:** Infectious diseases, Tuberculosis

## Abstract

Persons living with diabetes (PLWD) with newly diagnosed tuberculosis are at greater risk of poor treatment outcomes. Identifying and prioritizing high-risk subgroups of PLWD and tuberculosis for tuberculosis programs to target has been rarely performed. We investigated risk factors for poor tuberculosis treatment outcomes among PLWD and developed a predictive risk score for tuberculosis control prioritization. Among PLWD diagnosed with tuberculosis, demographic, clinical, and tuberculosis treatment outcome data were collected. Poor treatment outcomes included treatment failure, death, default, and transfer. Multivariable logistic regression modeling was used to analyze risk factors of poor treatment outcomes. Risk scores were derived based on regression coefficients to classify participants at low-, intermediate-, and high-risk of poor treatment outcomes. Among 335 PLWD newly diagnosed with tuberculosis, 109 were cured and 172 completed treatment. Multivariable logistic regression found that risk factors of poor treatment outcomes included bacteriologically-positivity, low body mass index, no physical activity, and pulmonary cavitation. Rates of poor treatment outcomes in low- (0–2), intermediate- (3–4), and high-risk (5–8) groups were 4.2%, 10.5%, and 55.4% (P_trend_ < 0.0001), respectively. The risk score accurately discriminated poor and successful treatment outcomes (C-statistic, 0.85, 95% CI 0.78–0.91). We derived a simple predictive risk score that accurately distinguished those at high- and low-risk of treatment failure. This score provides a potentially useful tool for tuberculosis control programs in settings with a double burden of both tuberculosis and diabetes.

## Introduction

Tuberculosis has been identified as one of the top 10 causes of death globally^1^. Persons with an impaired immune system, such as those living with diabetes, are at a higher risk of developing tuberculosis and having poor tuberculosis treatment outcomes once diagnosed^2–5^. Globally, the burden of diabetes is increasing at alarming rates—in 2019, there were 463 million (9.3%) persons living with diabetes (PLWD) which is predicted to rise to 10.2% (578 million) by 2030 and 10.9% (700 million) by 2045^6^. Currently, 80% of adults with diabetes reside in low- and middle-income countries where tuberculosis is also endemic^7,8^. An estimated 11% of all global tuberculosis deaths are attributable to diabetes^9^.

PLWD have an increased risk of poor tuberculosis treatment outcomes including failure and death during treatment and subsequent relapse^2–5,10,11^. Characteristics that put PLWD at-risk for treatment failure or death have been heterogeneous and how tuberculosis control programs can effectively and efficiently target PLWD newly diagnosed with tuberculosis for enhanced monitoring and investigation is not well elucidated^3^. Understanding subgroups of tuberculosis patients with diagnosed diabetes at highest risk of treatment failure is critical for tuberculosis control programs to prioritize enhanced management and resources for these patients.

We aimed to investigate tuberculosis treatment outcomes among a cohort of PLWD newly diagnosed with tuberculosis in Jiangsu province, China. We developed a risk classification model that may be clinically useful to identify and prioritize PLWD newly diagnosed with tuberculosis.

## Methods

### Study design and participants

The National Basic Public Health Service Project has been conducted since 2009 in China and provides basic public health services to the general public free of charge, focusing on children, pregnant women, elderly, and patients with chronic diseases. This service includes the management of PLWD age 35 and above with physical examinations performed twice a year. Participants of this study were based in four cities in Jiangsu Province: Danyang, Rugao, Jiangyin and Nanjing city, China. All diagnosed diabetes patients in care participated in the diabetes physical examination from January to December 2017. Physical examinations for type 2 diabetes include fasting blood glucose test, height, weight, waist circumference, body temperature, pulse, respiration, and blood pressure. We collected physical examination and anti-tuberculosis treatment outcome data among PLWD tuberculosis patients from the Chinese Tuberculosis Information Management System^12,13^. To further assess tuberculosis among PLWD, we used unique identification numbers from each patient to crossmatch the screening database of the Diabetes Physical Examination System and the Tuberculosis Management Information System. After linkage by the unique identification numbers, age, first and last name, date of birth, sex, and address were compared for confirmation. We then summarized the information of the matched patients. The research was performed in accordance with the Declaration of Helsinki.

### Study definitions

All diagnosed PLWD were screened for tuberculosis through a clinical symptom screen, chest X-rays, and bacteriological testing. Tuberculosis diagnosis was made based on definitions provided by the World Health Organization^14^. New tuberculosis cases were defined as tuberculosis patients whose medical records indicated that the patient had denied having any prior anti-tuberculosis treatment or any history of more than 30 days of anti-tuberculosis treatment. Previously treated tuberculosis cases were defined as persons with documented evidence of prior treatment in the case report or surveillance database. A 3HRZE/6HR treatment regimen was used for diabetes-tuberculosis patients based on recommendations by the World Health Organization and the National Tuberculosis Control Program, China^14,15^. Treatment outcomes were defined as designated by World Health Organization guidelines^16,17^. Cured was defined as a pulmonary tuberculosis patient with culture-confirmed tuberculosis at the beginning of treatment who was culture-negative in month 5 or 6 during anti-tuberculosis treatment. Cultures were obtained at two, five, and the end of anti-tuberculosis treatment; ‘treatment failure’ was recorded if cultures or smears were positive at the 5th and 6th month of treatment. Death comprised of any patient who deceased for any reason during the course of tuberculosis treatment. Poor treatment outcomes only included treatment failure, death and treatment interruption due to adverse reaction. Glycemic control among PLWD was defined as poor if a participant’s first fasting plasma glucose test was above either 7.2 (definition 1) or 10 mmol/L (definition 2), based on recommendations from the American Diabetes Association^18^ and prior studies^19,20^.

### Statistical analysis

Treatment outcome data and risk factors were analyzed and compared in the patient cohort. Descriptive analyses were performed to characterize distributions of available variables in our study population. Logistic regression analysis was used to analyze risk factors of tuberculosis treatment outcomes for tuberculosis patients with diabetes. We then derived risk scores through a multi-pronged approach. Briefly, univariable logistic regression was used to determine the relationship of each independent variable for poor treatment outcomes. In multivariable logistic regression model building, we selected variables with clinical significance and all predictors with a *P* value < 0.1 in the univariable model^21^. Sex and age were included in the multivariable logistic model regardless of *P* value. We then identified variables with statistically significant independent predictive value (*P* < 0.1) in the multivariable logistic regression model using Hosmer–Lemeshow model tests. We checked for linearity in the logit of continuous variables and for significant interaction terms^22^. We also assessed goodness of fit and stability during model building. Odds ratio (OR) and 95% confidence intervals (CI) were calculated and used to describe the impact of related factors on treatment outcomes of diagnosed diabetes patients with tuberculosis.

We then derived risk scores based on multivariable logistic regression models. We followed previously published recommendations for developing predictive risk scores^23,24^. Briefly, we computed how far each subcategory of a risk factor was from the base category for each predictor variable in the multivariable analysis and derived a constant for the points system relating to the number of regression units corresponding to 1 point. The risk score model is derived to compute the required ∑βX for a given risk factor profile. The risk estimate was then determined from a reference table which provides risk estimates for each point total. While the function itself can accommodate distinct values for the risk factors (e.g., age, sex, body mass index) on a continuous scale. The points system is organized around categories in order to mirror clinically meaningful risk factor states^24^.

We assigned a point score based on a transformation of corresponding β regression coefficients. The subsequent score was rounded to the nearest integer for clinical and programmatic practicality. We then calculated a risk score for each individual patient. The study population was grouped into three risk stratifications (low-, intermediate-, and high-risk) based on the probability of poor treatment outcomes in each group. Receiver operating characteristic curve analyses for the risk score was performed to assess the performance of the score. We internally validated the risk score using tenfold cross-validation. All statistical analyses were conducted using SPSS software (version 23.0).

### Ethics statement

This project was approved by Institutional Review Board of Jiangsu Provincial Center for Disease Control and Prevention. Written informed consent was obtained from all participants.

### Consent for publication

All co-authors consent to this submission.

## Results

### Demographic characteristics

**Figure 1:**
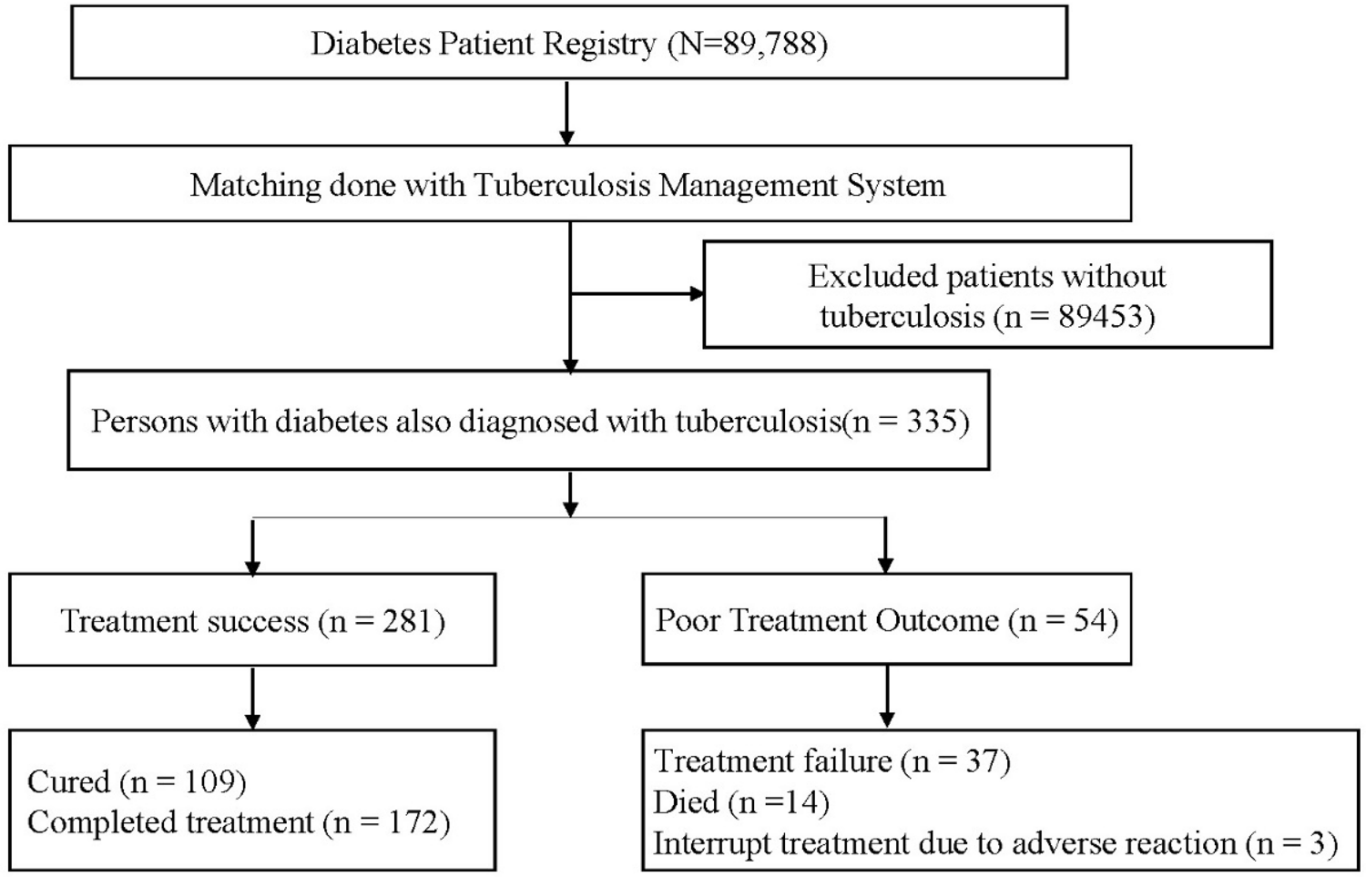
Flowchart for the treatment outcomes of tuberculosis patients with diabetes after treatment completed in Jiangsu province, China.

The National Basic Public Health Service Diabetes Patients Physical Examination Project were conducted in four cities (Danyang, Rugao, Jiangyin, and Nanjing). A total of 335 PLWD and tuberculosis were included in this study (Fig. 1). The mean age was 64.9 (Standard Deviation, ± 11.9) years old and 234 (69.9%) were male. Most patients did not drink (79.1%) or smoke (71.6%). Chest radiographs and sputum smear were performed in all patients, 28.5% (96/337) patients had lung cavities, 57.3% (193/335) were smear-negative, and 14.9% (N = 50) were previously treated tuberculosis patients. Among smear-positive patients, the sputum smear conversion rate was 77.1% (N = 111) after intensive treatment (Table 1). Of 335 patients, 109 were cured and 172 completed treatment. In all, 54 tuberculosis cases (16.11%) had poor treatment outcomes. Of these, 14 died (25.9%), 37 (68.5%) failed treatment, and 3 (5.6%) had treatment interruption due to adverse events (Fig. 1).

**Table 1:** Characteristics of 337 Persons Living with Diabetes Diagnosed with Tuberculosis in Eastern China.

Variable	Total, N (%)	Treatment success, N (%)	Poor outcomes, N (%)	*P*-value
All	335 (100)	281 (83.9)	54 (16.1)	
Mean age (± SD), years	64.90 ± 11.90	64.96 ± 11.40	64.56 ± 14.31	
**Sex**				0.0073
Male	234 (69.9)	188 (66.9)	46 (85.2)	
Female	101 (30.1)	93 (33.1)	8 (14.8)	
**City**				0.0000
Jiangyin	115 (34.3)	106 (37.7)	9 (16.7)	
Danyang	49 (14.6)	41 (14.6)	8 (14.8)	
Rugao	89 (26.6)	80 (28.5)	9 (16.7)	
Nanjing	82 (24.5)	54 (19.2)	28 (51.9)	
**Body mass index, kg/m**^**2**^				0.0080
< 18.5	18 (5.4)	10 (3.6)	8 (14.8)	
18.5–23.9	174 (51.9)	147 (52.3)	27 (50.0)	
24.0–26.9	96 (28.7)	82 (29.2)	14 (25.9)	
≥ 27.0	47 (13.9)	42 (14.9)	5 (9.3)	
Smoking History*				0.2243
Yes	95 (28.4)	76 (27.0)	19 (35.2)	
No	240 (71.6)	205 (73.0)	35 (64.8)	
Drinking history*				0.9175
Yes	70 (20.9)	59 (21.0)	11 (20.4)	
No	265 (79.1)	222 (79.0)	43 (79.6)	
Physical activity, Exercise*				0.0000
No	233 (69.6)	183 (65.1)	50 (92.6)	
Yes	102 (30.4)	98 (34.9)	4 (7.4)	
**Lung Cavitation**				0.0000
Yes	96 (28.7)	59 (21.0)	37 (68.5)	
No	239 (71.3)	222 (79.0)	17 (31.5)	
**Sputum smear**				0.0000
Positive	144 (43.0)	105 (37.4)	39 (72.2)	
Negative	191 (57.0)	176 (62.6)	15 (27.8)	
**Bacteriological#**				0.0000
Positive	168 (50.1)	123 (43.8)	45 (83.3)	
Negative	167 (49.9)	158 (56.2)	9 (16.7)	
**Treatment history**				0.0010
New	285 (85.1)	247 (87.9)	38 (70.4)	
Retreated	50 (14.9)	34 (12.1)	16 (29.6)	
Fasting blood glucose, mmol/L	8.98 ± 4.07	8.76 ± 3.42	10.14 ± 6.41	0.0300
**Glycemic control, 7.2 mmol/L‡**				0.536
Good	124 (37.0)	102 (36.3)	22 (40.7)	
Poor	211 (63.0)	179 (63.7)	32 (59.3)	
**Glycemic Control, 10 mmol/L‡**				0.2860
Good	249 (74.3)	212 (75.4)	37 (68.5)	
Poor	86 (25.7)	69 (24.6)	17 (31.5)	
**Hypoglycemic drugs**				0.6570
Yes	76 (22.7)	65 (23.1)	11 (20.4)	
Missing	259 (77.3)	216 (76.9)	43 (79.6)	

*Physical Activity, Smoking History and Drinking History were self-reported, including currently or past. Bacteriological#, including the result of sputum culture or smear examination.

^‡^Glycemic control among persons living with diabetes was defined as poor if a participant’s first fasting plasma glucose test was above either 7.2 or 10 mmol/L.

### Risk factors for poor tuberculosis treatment outcomes

In univariable logistic regression analyses, men were more likely to experience poor treatment outcomes (OR, 2.8, 95% CI 1.3–6.3). Poor treatment outcomes were also more likely when patients were bacteriologically-positive (OR, 6.4, 95% CI 3.0–13.6), had pulmonary cavitation (OR, 8.2, 95% CI 4.3–15.6), had a previous tuberculosis episode (OR, 3.1, 95% CI 1.5–6.1), no exercise (OR, 6.7, 95% CI: 2.4–19.1), and patients with a body mass index < 18.5 (OR, 4.4, 95% CI 1.6–12.0 compared to participants with a body mass index between 18.5 and 23.9) (Table 2).

**Table 2:** Risk factors for poor treatment outcomes in persons living with diabetes and tuberculosis in eastern China.

Variable	Univariable analysis			Multivariable analysis		
	*P*-value	Odds Ratio	95% CI	*P*-value	Odds Ratio	95% CI
Age (continuous), years	0.8200	1.00	0.97–1.02	0.9260	1.00	0.97–1.03
**Sex**						
Female		1 (Referent)			1 (Referent)	
Male	0.0100	2.84	1.29–6.27	0.1040	2.16	0.85–5.48
**Body mass index, kg/m**^**2**^						
18.5–23.9		1 (Referent)			1 (Referent)	
< 18.5	0.0050	4.36	1.58–12.03	0.0040	6.66	1.83–24.27
24.0–26.9	0.8380	0.93	0.46–1.87	0.8990	1.06	0.46–2.45
≥ 27.0	0.4020	0.65	0.24–1.79	0.6710	1.30	0.39–4.29
Physical activity, exercise*						
Yes		1 (Referent)			1 (Referent)	
No	0.0000	6.70	2.35–19.08	0.0020	5.96	1.93–18.45
**Lung cavitation**						
No		1 (Referent)			1 (Referent)	
Yes	0.0000	8.19	4.31–15.56	0.0000	6.05	2.82–12.98
**Bacteriological#**						
Negative		1 (Referent)			1 (Referent)	
Positive	0.0000	6.42	3.02–13.64	0.0050	3.37	1.44–7.92
**Treatment history**						
New		1 (Referent)			1 (Referent)	
Retreated	0.0000	3.06	1.54–6.07	0.0620	2.22	0.96–5.15
Fasting blood glucose, mmol/L	0.0300	1.07	1.01–1.14	0.4960	1.03	0.95–1.11

*BMI, Body mass index, taking into account factors such as differences in the diet and physique of Chinese and foreigners, BMI of this study is classified by Chinese standards, including < 18.5 was thin, 18.5–23.9 was normal level, 24.0–26.9 was overweight and ≥ 27.0 was obesity. Physical Activity, Exercise, including walking, running or other forms of exercise. Bacteriological#, including the result of sputum culture or smear examination.

In a multivariable logistic regression analysis, similar characteristics were risk factors for poor treatment outcomes. These included a low body mass index < 18.5 (Adjusted Odds Ratio [AOR], 6.7, 95% CI 1.8–24.3), no exercise (AOR, 6.0, 95% CI 1.9–18.5), bacteriological positivity (AOR, 3.4, 95% CI 1.4–7.9), and pulmonary cavitation (AOR, 6.1, 95% CI 2.8–13.0) (Table 2).

### Development of predictive risk score

**Figure 2:**
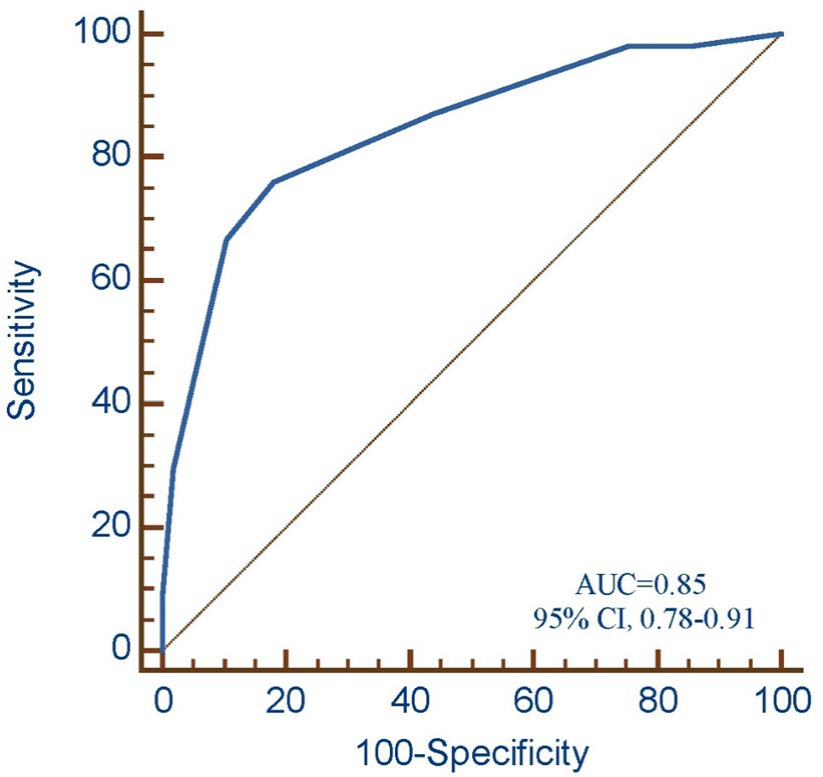
Receiver operating characteristic curve analysis for the risk scores in tuberculosis patients with diabetes.

When assigning a point score, patients were assigned 1 point if they had a previous tuberculosis episode or were bacteriologically positive. Patients with lung cavitation, a body mass index < 18.5, or that did not do any physical activity were given a total of 2 points (Supplementary Table 1). The score ranged from 0 to 8 and treatment failure rates increased with higher score (P_trend_ < 0.0001). The estimated risk of poor treatment outcomes increased from 0.3% in patients with 0 points to 60.8% among patients with a score of 8 (Supplementary Table 2). After grouping patients into low- (0–2 points), intermediate- (3–4 points), and high-risk (5–8 points) classification groups, most treatment failures (66.7, 36/54) occurred in the high-risk group and 87.0% (48/54) occurred in intermediate- and high-risk groups. Treatment failure rates were 4.2% (95% CI 1.1–7.4), 10.5% (95% CI 4.4–16.1), and 55.4 (95% CI , 43.0–67.8) in low-, intermediate-, and high-risk groups, respectively (P_trend_ < 0.0001). The absolute difference in probability of treatment failure between high- and low-risk groups was 51.2% in the cohort (Table 3). The risk score discriminated adverse and successful treatment outcomes well (AUC, 0.85, 95% CI 0.78–0.91) (Fig. 2). In an internal tenfold cross-validation, the risk score had strong predictive ability with a C statistic of 0.83 (95% CI 0.74–0.89).

**Table 3:** Risk of poor outcomes among persons living with diabetes diagnosed with tuberculosis according to risk category.

	Risk category			All Participants
	Low (< 3 points)	Intermediate (3–4 points)	High (≥ 5 points)	
No., %	165 (49.3)	105 (31.3)	65 (19.4)	335 (100)
Poor Outcomes, % (95% CI)	4.2 (1.1–7.4)	10.5 (4.4–16.1)	55.4 (43.0–67.8)	16.1 (12.2–20.1)
Odds Ratio (95% CI), *P*-value	1 (Referent)	2.56 (0.98–6.72), 0.056	30.96 (12.93–74.15), 0.000	…
Risk Difference, % (95% CI)	1 (Referent)	6.3 (3.3–8.7)	51.2 (41.9–60.4)	…
C–statistic (95% CI)‡	…	…	…	0.85 (0.78–0.91)
**10-fold Cross-Validation**				
Optimism-adjusted C–statistic (95% CI)‡*	…	…	…	0.83 (0.74–0.89)
All Poor Outcomes in High, %	…	…	…	66.7
All Poor Outcomes in High and Intermediate, %	…	…	…	87.0

Risk Difference, this is the percent difference between the intermediate- and high-risk group versus the low-risk group.

## Discussion

PLWD diagnosed with tuberculosis are at high-risk of treatment failure and death^2–4,25^. However, identifying PLWD at highest risk of treatment failure is critical for tuberculosis control programs with a high diabetes prevalence, such as China. Effective and straightforward tools for public health professionals are urgently needed. We found that PLWD and tuberculosis that were bacteriologically positive, with low body mass index (< 18.5), and with low levels of physical activity were more likely to experience poor treatment outcomes. We derived a prognostic score to ranging from 0–8 which predicted well with treatment failure and death and may be useful for programmatic implementation in tuberculosis control programs.

These results suggest that identifying tuberculosis patients at high- and low probability of poor outcome may be feasible using a set of commonly collected patient-level factors. The risk score had a strong degree of accuracy to discriminate between adverse and successful treatment outcomes (C-statistic, 0.85). There may be unmeasured confounding and data quality may be suboptimal. However, our study, which uses routinely collected data by National Basic Public Health Service Diabetes Patients Physical Examination Project and national tuberculosis programs, has an important ‘real-world’ context and may be practically used to PLWD without additional costs. Additional studies with further risk factors may be useful to improve the diagnostic accuracy of this prognostic model.

Patients that did not exercise had an increased risk of poor outcomes in PLWD diagnosed with tuberculosis, suggesting physical activity may significantly reduce risk of adverse outcomes. This may be related to case severity. If hospitalized patients, who are often the most severe, are unable to exercise this may explain this relationship. Low body mass index (< 18.5) also increased the risk of adverse treatment outcomes. This has been shown in previous studies^25,26^. Persons with a low body mass index or nutritional disequilibrium may alter the host immune response leading to more severe forms of tuberculosis impacting subsequent poor treatment outcomes^27^.

We found that patients with bacteriologically positive results at baseline were also more likely to experience poor outcomes. In this study, 82% of patients with poor treatment outcomes were bacteriologically positive, of which 39 were sputum smear positive. Our study did not demonstrate an association of alcohol consumption and smoking with poor treatment outcomes. Other determinants of poor treatment outcomes among tuberculosis patients include lifestyle factors such as smoking, alcohol consumption and drug abuse^2,28^. There are various mechanisms through which smoking may adversely impact tuberculosis treatment outcome, for example through altering immunological host defense mechanisms, impacting lung structure and function, and modifying mechanisms of pathogen clearance^28^. This lack of association may be attributed to small numbers of self–reported alcohol consumption and smoking, likely an underestimate considering the social, cultural and religious norms that exist in China^29^. Importantly, we found that glycemic control did not impact tuberculosis treatment outcomes. Prior studies among participants both with and without diabetes found that glycemic control among PLWD was a risk factor for poor treatment outcomes compared to participants without diabetes^3,4^. However, our study included only PLWD, therefore the reference group is PLWD with poor glycemic control (rather than persons without diabetes), likely explaining a lack of association with poor treatment outcomes in our study.

There are limitations to this study. First, we had substantial missing data on hypoglycemic drugs used by participants limiting our ability to study the effect of diabetes drug use on tuberculosis treatment outcomes. Poor treatment outcomes may be mediated through specific drugs, such as metformin and, due to this, our results may be impacted. However, drug use may be related to glucose control which was measured in our study. Second, although we included a large number of diagnosed diabetes/tuberculosis patients in the province, our sample size was < 400 patients and a larger sample size may have elucidated further risk factors for poor tuberculosis treatment outcomes. The diagnosis of diabetes was not based on blood glucose levels alone; we enrolled subjects who were known to have diabetes to rule out bias associated with transient hyperglycemia attributed to tuberculosis^4,30^. Lastly, we were not able to externally validate our prognostic model in another setting which is necessary prior to implementation of such a score in a public health program. Because we did not include a validation cohort from outside of China, it is unclear how these results are generalizable to persons with diabetes/tuberculosis in other countries with large dual epidemics.

### Conclusions

Our study shows that PLWD with tuberculosis experienced treatment failure more commonly when bacteriologically positive, previous tuberculosis treatment, low body mass index, limited physical activity, or lung cavitation. Integrated models of care with early screening and management for diabetes and tuberculosis should be initiated. The detection of diabetes in tuberculosis patients and linking these persons to care may improve tuberculosis treatment outcomes. Strengthening early diagnosis and identifying tuberculosis patients with diabetes that are at high-risk of poor treatment outcomes is needed in areas with a high burden of both diabetes and tuberculosis.

## Supplementary Information

Supplementary Information 1.

Supplementary Information 2.

## Data Availability

Please contact the first author for data requests.

## Abbreviations

PLWD: Persons living with diabetes
CI: Confidence intervals
OR: Odds ratio
AOR: Adjusted odds ratio
