# Supplementary material for: A risk score for prediction of poor treatment outcomes among tuberculosis patients with diagnosed diabetes mellitus from eastern China
Source: Sci Rep. 2021 May 27;11:11219. doi: 10.1038/s41598-021-90664-y (PMC8160203; doi:10.1038/s41598-021-90664-y)
Supplement: Supplementary file 1 — Supplementary Information 1. [file 41598_2021_90664_MOESM1_ESM.docx]

**Supplementary Appendix**

Supplement to: A Risk Score for Prediction of Poor Treatment Outcomes among Tuberculosis

Patients with Diagnosed Diabetes Mellitus from Eastern China

Nannan You^1^, Hongqiu Pan^2^, Yi Zeng^3^, Peng Lu^1^,

Limei Zhu^1^, Wei Lu^1^, Qiao Liu^1^, Leonardo Martinez^4^

1. Department of Chronic Communicable Disease, Center for Disease Control and Prevention of Jiangsu Province, Nanjing, Jiangsu Province, PR China

2. Department of Tuberculosis, The Third People's Hospital of Zhenjiang Affiliated to Jiangsu University, Zhenjiang, China

3. Department of Tuberculosis of Three, Nanjing Public Health Medical Center, Nanjing Second Hospital, Nanjing Hospital Affiliated to Nanjing University of Traditional Chinese Medicine, Nanjing, Jiangsu Province, PR China

4. Department of Epidemiology, School of Public Health, Boston University, Boston, Massachusetts, United States

Nannan You and Hongqiu Pan contributed equally to this work.

Tables and Figures.

Supplementary Table 1. The scores of risk factors for poor treatment outcomes in diabetic patients with tuberculosis†

Supplementary Table 2. Point total and risk estimate for poor tuberculosis treatment outcomes among persons living with diabetes diagnosed with tuberculosis in eastern China

Supplementary Table 1. The scores of risk factors for poor treatment outcomes in diabetic patients with tuberculosis†

| **Risk factors** | **Categories** | **Reference value (Wij)** | **βi** | **βi (Wij–WiREF)** | **Pointsij= βi(Wij–WiREF)/B** |
| --- | --- | --- | --- | --- | --- |
| Treatment History | |  | 0.7160 |  |  |
|  | New patients | 0=WiREF |  | 0.0000 | 0 |
|  | Retreated patients | 1 |  | 0.7160 | 1 |
| Bacteriological | |  | 1.1910 |  |  |
|  | Negative | 0=WiREF |  | 0.0000 | 0 |
|  | Positive | 1 |  | 1.1910 | 1 |
| Lung Cavitation | |  | 1.6630 |  |  |
|  | No | 0=WiREF |  | 0.0000 | 0 |
|  | Yes | 1 |  | 1.6630 | 2 |
| Physical Activity, Exercise | |  | 1.9130 |  |  |
|  | Yes | 0=WiREF |  | 0.0000 | 0 |
|  | No | 1 |  | 1.9130 | 2 |
| BMI<18.5 |  |  | 1.7090 |  |  |
|  | No | 0=WiREF |  | 0.0000 | 0 |
|  | Yes | 1 |  | 1.7090 | 2 |

† Wij, the risk factors; WiREF, the referent risk factors’ value; βi, was regression coefficient of multivariable logistic regression analysis; B, we define the constant for the points system was 1 for easily calculate; Bacteriological#, including the result of sputum culture or smear examination. Physical Activity, Exercise, including walking, running or other forms of exercise.

Supplementary Table 2. Point total and risk estimate for poor tuberculosis treatment outcomes among persons living with diabetes diagnosed with tuberculosis in eastern China

| **Point total** | **Estimate of risk**† | **N (%)** | **No. of poor outcomes (%)** | **95%CI** |
| --- | --- | --- | --- | --- |
| 0 | 0.0054918 | 41 (12.2) | 1(2.4) | -0.025-0.074 |
| 1 | 0.0147886 | 29 (8.6) | 0(0) | / |
| 2 | 0.0392034 | 96 (28.5) | 7(7.3) | 0.020-0.126 |
| 3 | 0.0998403 | 79 (23.4) | 7(8.9) | 0.025-0.153 |
| 4 | 0.2316532 | 27 (8.0) | 5(18.5) | 0.029-0.342 |
| 5 | 0.4504135 | 44 (13.1) | 20(45.5) | 0.301-0.608 |
| 6 | 0.6901884 | 16 (4.7) | 11(68.8) | 0.432-0.943 |
| 7 | 0.8582706 | 5 (1.5) | 5(100) | / |
| 8 | 0.9427298 | 0 (0) | 0(0) | / |

† Estimate of risk, is risk estimate to each point using the multiple logistic regression equation as described in the Methods and Results sections.
