# Supplementary material for: A risk score for prediction of poor treatment outcomes among tuberculosis patients with diagnosed diabetes mellitus from eastern China
Source: Sci Rep. 2021 May 27;11:11219. doi: 10.1038/s41598-021-90664-y (PMC8160203; doi:10.1038/s41598-021-90664-y)
Supplement: Supplementary file 2 — Supplementary Information 2. [file 41598_2021_90664_MOESM2_ESM.docx]

Supplementary Table 1. The scores of risk factors for poor treatment outcomes in diabetic patients with tuberculosis†

| **Risk factors** | **Categories** | **Reference value (Wij)** | **βi** | **βi (Wij–WiREF)** | **Pointsij= βi(Wij–WiREF)/B** |
| --- | --- | --- | --- | --- | --- |
| Treatment History | |  | 0.7990 |  |  |
|  | New patients | 0=WiREF |  | 0.0000 | 0 |
|  | Retreated patients | 1 |  | 0.7990 | 1 |
| Bacteriological | |  | 1.2160 |  |  |
|  | Negative | 0=WiREF |  | 0.0000 | 0 |
|  | Positive | 1 |  | 1.2160 | 1 |
| Lung Cavitation | |  | 1.8000 |  |  |
|  | No | 0=WiREF |  | 0.0000 | 0 |
|  | Yes | 1 |  | 1.8000 | 2 |
| Physical Activity, Exercise | |  | 1.7850 |  |  |
|  | Yes | 0=WiREF |  | 0.0000 | 0 |
|  | No | 1 |  | 1.7850 | 2 |
| BMI<18.5 |  |  | 1.8950 |  |  |
|  | No | 0=WiREF |  | 0.0000 | 0 |
|  | Yes | 1 |  | 1.8950 | 2 |

† Wij, the risk factors; WiREF, the referent risk factors’ value; βi, was regression coefficient of multivariable logistic regression analysis; B, we define the constant for the points system was 1 for easily calculate; Bacteriological#, including the result of sputum culture or smear examination. Physical Activity, Exercise, including walking, running or other forms of exercise.

Supplementary Table 2. Point total and risk estimate for poor tuberculosis treatment outcomes among persons living with diabetes diagnosed with tuberculosis in eastern China

| **Point total** | **Estimate of risk**† | **N (%)** | **No. of poor outcomes (%)** | **95%CI** |
| --- | --- | --- | --- | --- |
| 0 | 0.003043 | 41 (12.2) | 1(2.4) | 0.0013-0.1440 |
| 1 | 0.006607 | 29 (8.7) | 0(0) | / |
| 2 | 0.014287 | 95 (28.4) | 6(6.3) | 0.0259-0.1377 |
| 3 | 0.030620 | 78 (23.3) | 6(7.7) | 0.032-0.1659 |
| 4 | 0.064404 | 27 (8.1) | 5(18.5) | 0.070-0.3875 |
| 5 | 0.130448 | 44 (13.1) | 20(45.5) | 0.3069-0.6100 |
| 6 | 0.246382 | 16 (4.8) | 11(68.8) | 0.4148-0.8787 |
| 7 | 0.416052 | 5 (1.5) | 5(100) | / |
| 8 | 0.608259 | 0 (0) | 0(0) | / |

† Estimate of risk, is risk estimate to each point using the multiple logistic regression equation as described in the Methods and Results sections.
